# Intramolecular Modulation of Serine Protease Inhibitor Activity in a Marine Cyanobacterium with Antifeedant Properties

**DOI:** 10.3390/md8061803

**Published:** 2010-06-04

**Authors:** Susan Matthew, Ranjala Ratnayake, Mikel A. Becerro, Raphael Ritson-Williams, Valerie J. Paul, Hendrik Luesch

**Affiliations:** 1 Department of Medicinal Chemistry, University of Florida, 1600 SW Archer Road, Gainesville, FL 32610, USA; E-Mails: susmatt@ufl.edu (S.M.); rratnayake@ufl.edu (R.R.); 2 Smithsonian Marine Station, 701 Seaway Drive, Fort Pierce, FL 34949, USA; E-Mails: mikel@ceab.csic.es (M.A.B.); williams@si.edu (R.R.-W.)

**Keywords:** cyanobacteria, Lyngbya, antifeedant activity, serine protease inhibitors, cyclodepsipeptides

## Abstract

Extracts of the Floridian marine cyanobacterium *Lyngbya* cf. *confervoides* were found to deter feeding by reef fish and sea urchins (*Diadema antillarum*). This antifeedant activity may be a reflection of the secondary metabolite content, known to be comprised of many serine protease inhibitors. Further chemical and NMR spectroscopic investigation led us to isolate and structurally characterize a new serine protease inhibitor **1** that is formally derived from an intramolecular condensation of largamide D (**2**). The cyclization resulted in diminished activity, but to different extents against two serine proteases tested. This finding suggests that cyanobacteria can endogenously modulate the activity of their protease inhibitors.

## 1. Introduction

**Figure 1:**
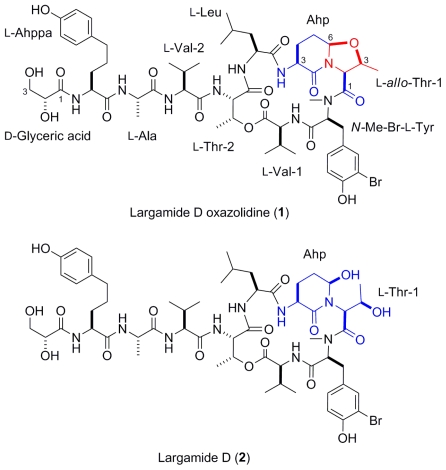
Structures of largamide D oxazolidine (**1**) and the parent compound, largamide D (**2**).

Both marine and freshwater cyanobacteria contain toxic secondary metabolites and are notorious for their ability to form extensive blooms. The compounds found in these bloom-forming cyanobacteria include the lyngbyatoxins and the hepatotoxic microcystins and cylindrospermopsin [1–6]. Several cyanobacterial extracts can reduce feeding by fish, which is thought to be driven by secondary metabolite diversity and abundance [6–9]. However, cyanobacteria also produce a range of protease inhibitors with little or no apparent cytotoxicity. One prevalent class found in marine [10–13] and freshwater [14–16] cyanobacteria is comprised of protease inhibitors with a cyclic depsipeptide scaffold that contains a 3-amino-6-hydroxy-2-piperidone (Ahp) moiety as a key feature for inhibition of certain serine proteases. Since many digestive enzymes such as trypsin and chymotrypsin are serine proteases and are inhibited by these compounds, these natural products could function as digestion inhibitors [17]. Serine protease inhibitors also co-occur with microcystins and are linked to an enhanced toxin activity [18] or thought to upregulate biosynthetic genes [19]. Here we investigate the antifeedant activity of a *Lyngbya* species identified as *Lyngbya* cf. *confervoides* (cf. = similar to but not the same as; compared to *Lyngbya confervoides*) [10,20] collected from reefs near Fort Lauderdale, Florida, towards natural assemblages of reef fish and the tropical sea urchin *Diadema antillarum*. This cyanobacterium produces a wide array of serine protease inhibitors including lyngbyastatins 4–6 [21,22], pompanopeptin A [23] and largamides D–G [24]. Extracts of this cyanobacterium deterred fish and urchin feeding, which may be linked to the production of these protease inhibitors, however, individual compounds have not been tested for their ability to deter feeding. This observed feeding deterrent activity led us to thoroughly investigate the natural product chemistry of this cyanobacterium. Through rigorous chemical investigation of the crude extracts we identified a new compound, largamide D oxazolidine (**1**), which is an intramolecular condensation product of the serine protease inhibitor largamide D (**2**) (Figure 1). Largamide D oxazolidine (**1**) exhibited a reduced and differential activity against chymotrypsin and elastase, providing evidence for an endogenous mode of deactivation or modulation of protease inhibitor activity in this marine cyanobacterium.

## 2. Results and Discussion

### 2.1. Feeding Experiments

The extractions yielded a dry weight concentration of 21.15% for the non-polar extract and 6.95% for the polar extract. All feeding experiments were conducted at natural concentration by dry weight.

**Figure 2:**
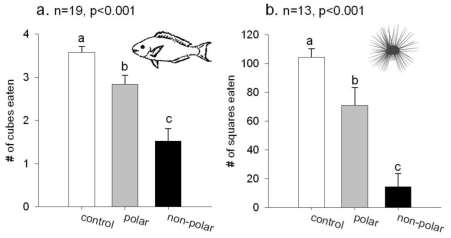
Feeding experiments with the crude extracts of *L.* cf. *confervoides*. The bars represent the mean amount of food eaten and the error bars are +1 SE. Different letters above the bars represent means that are statistically different. (**a**). Feeding experiments with a natural assemblage of reef fish. (**b**). Feeding experiments with the sea urchin *Diadema antillarum*.

When tested against a natural assemblage of reef fish in field feeding experiments, where many individuals of diverse fish species would feed on food cubes containing cyanobacterial extracts, both the polar and non-polar extracts reduced feeding on agar food cubes. The fish consumed 3.58 ± 0.14 (mean ± SE) agar cubes of the control food, 2.84 ± 0.21 agar cubes of the food containing the polar extract, and 1.52 ± 0.29 of the agar cubes that contained the non-polar extract (Figure 2a). There were significant differences in the amount of food eaten (n = 19, Friedman’s random block, p < 0.001), with less of the polar extract eaten by the fish than the solvent controls and the least amount eaten of the food containing the non-polar extract (Student Newman Keuls post-hoc test, p < 0.05).

When extracts of *L.* cf. *confervoides* were tested against the sea urchin *Diadema antillarum*, both the polar and non-polar extracts reduced urchin feeding. The urchins ate 104.3 ± 6.0 (mean ± SE) squares of the control food, 71.0 ± 12.3 squares of the food with the polar extract and 14.3 ± 9.3 squares of the food with the non-polar extract (Figure 2b). There were statistically different amounts of the food consumed (n = 13, Friedman’s random block, p < 0.001) with the solvent control the most consumed food, less eaten of the food with the polar extract and the least eaten of the food with the non-polar extract (Student Newman Keuls post-hoc test, p < 0.05).

Other benthic marine cyanobacteria have also been shown to be chemically defended from marine herbivores [6–9,25]. Ypaoamide, pitipeptolide A, malyngolide and malyngamides A and B are a few purified compounds from marine cyanobacteria known to reduce feeding by generalist herbivores such as fishes and sea urchins [7,26,27]. More specialized herbivores, such as the sea hare *Stylocheilus striatus*, are usually not deterred by cyanobacterial extracts or compounds [6,9,26], and extracts of *L.* cf. *confervoides* also did not deter this herbivore relative to controls [28]. However, *Stylocheilus striatus* grew more slowly on *L.* cf. *confervoides* relative to *L. polychroa*, which might be related to the presence of serine protease inhibitors in *L.* cf. *confervoides* that reduced conversion efficiency of this diet [28].

### 2.2. Isolation and Structure Determination of Largamide D Oxazolidine (**1**)

**Figure 3:**
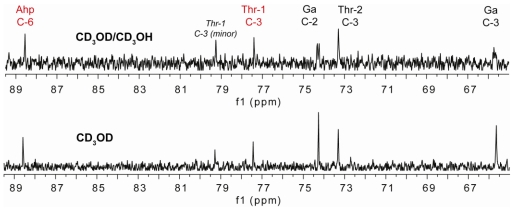
Expanded regions of the ^13^C NMR spectra (125 MHz) of **1** in CD_3_OD (bottom panel) and 1:1 CD_3_OD/CD_3_OH (top) showing peak splitting for carbons attached to the free hydroxyl groups.

The organic extracts from separate samples of the marine cyanobacterium *L.* cf. *confervoides* collected from two different locations from reefs near Ft. Lauderdale (Florida, USA) were subjected to HP-20 fractionation and subsequent HPLC purification to yield either largamide D (**2**) [24] or a new intramolecular condensation product, largamide D oxazolidine (**1**) (Figure 1). The structure of **2** was established by comparison with reported data [24]. Compound **1** displayed a pseudomolecular ion [M + Na]^+^ peak at *m/z* 1236.4745 and an [M + 2 + Na]^+^ isotope peak of similar intensity characteristic for bromine, which was consistent with a molecular formula of C_56_H_80_BrN_9_NaO_16_. The ^1^H and ^13^C NMR spectra (Table 1), featuring typical signals for a peptide, were suggestive of a close analog of largamide D (**2**). A detailed analysis of the 2D NMR (COSY, HSQC, HMBC and TOCSY) spectral data of **1** in DMF-*d*_7_ clearly supported the structural fragments and enabled the connectivity of the depsipeptide along with the side chain, similar to **2**. The major chemical shift differences between **1** and **2** were observed around the Ahp and Thr-1 units. The remaining ^1^H and ^13^C NMR data of **1** in MeOH-*d*_4_ (Table 1) were in close agreement with the reported data for **2** [24]. Most pronounced were the ~11 ppm downfield shifts relative to **2** of the signals for C-6 of the Ahp unit (*δ*_C_ 88.6) and C-3 of Thr-1 (*δ*_C_ 77.4), indicative of alkylation and, to be in agreement with the molecular formula, consistent with a formal condensation of Ahp and Thr-1 resulting in an oxazolidine and overall in a fused bicyclic system. A ^13^ A ^13^C NMR experiment carried out in a mixture of CD_3_OD/CD_3_OH clearly showed doubling of signals attributable to carbons attached to the hydroxyl groups in glyceric acid (Ga, *δ*_C_ 74.2 and 65.6). Singlets at *δ*_C_ 88.6 (C-6, Ahp) and 77.4 (C-3, Thr-1) supported the absence of exchangeable protons in both Ahp and Thr-1 where cyclization has occurred (Figure 3). A second set of resonances in a solvent-dependent ratio (1:0.3 in DMF-*d*_7_ and 1:0.7 in MeOH-*d*_4_) was observed in the NMR spectra, indicating the presence of conformers.

**Figure 4:**
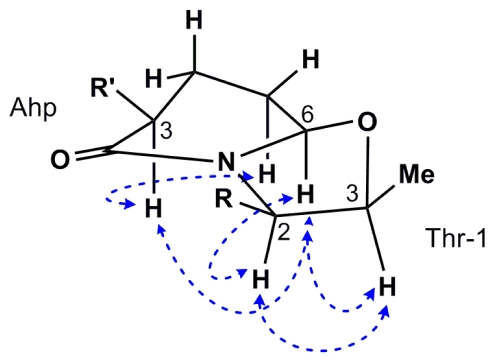
Selected key ROESY correlations among Ahp and Thr-1 for **1**.

Based on the comparison of the ^1^H and ^13^C NMR data between **1** and **2** in methanol, we propose the same configuration for **1** as reported for largamide D (**2**) [24] for all unchanged residues. The oxazolidine heterocycle formation apparently forced the Ahp unit to adopt a twisted boat-like conformation (Figure 4) as evidenced by the ROESY correlations of H-3 with H-5b and H-6, all of which had to be in pseudo-axial position on the same face of the ring. This conclusion is consistent with the observed coupling constants for H-3 (12.4 and 4.9 Hz) and two relatively large coupling constants for H-6 (8.5 and 6.2 Hz) which may be rationalized in this conformation (Figure 4). Assuming **1** retained the same configuration at C-3 of the Ahp as in **2—**since it is unaffected by cyclization—we propose a 3*S*,6*R* configuration for Ahp, which is identical to largamide D (**2**) [24]. Furthermore, ROESY correlations from Ahp H-6 to H-2 and H-3 of Thr-1 indicated that they are placed on the same side of the oxazolidine ring, providing evidence for a 2*S*,3*S* configuration for former Thr-1. Since the configuration of Thr-1 has been established as 2*S*,3*R* in **2**, the configuration at C-3 is inverted in compound **1** and represents the only configurational change compared with largamide D (**2**).

The inversion of C-3 configuration (Thr-1) suggests that the OH group of Ahp in **2** may have acted as a nucleophile to form the oxazolidine via nucleophilic substitution at C-3 of Thr-1 (rather than the opposite way), and this event could have been preceded by addition of an unknown leaving group at C-3 or the 3-OH group. Alternatively, Thr-1 could have been dehydrated to a 2-amino-2-butenoic acid (Abu) as encountered in lyngbyastatins, also produced by this cyanobacterium, which then served as a Michael acceptor, while the chiral environment induced the formation of a single diastereomer, compound **1**. While it is possible that compound **1** is derived from **2** as an isolation artifact, it is tempting to postulate that **1** is a plausible biosynthetic product. In favor of this assumption we note that **1** was not found in the samples of *L.* cf. *confervoides* that yielded largamide D (**2**) and *vice versa* from the two different collection sites. To the best of our knowledge this fused oxazolidine-containing bicyclic system is unprecedented in cyanobacteria but is found in diterpene alkaloids from *Spiraea japonica* [29] and in several polyketide compounds from *Actinomycete* spp. [30–32].

### 2.3. Serine Protease Inhibition Study

**Figure 5:**
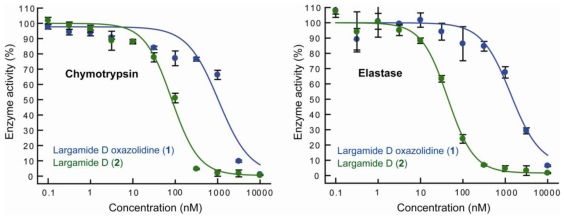
Effect of compounds **1** and **2** on chymotrypsin and elastase activity.

The presence of an Ahp unit is characteristic for many serine protease inhibitors, including lyngbyastatins. Largamide D (**2**) was previously reported to be a moderate chymotrypsin inhibitor [24]. We directly compared the activities of compound **1** and largamide D (**2**) against two serine proteases, chymotrypsin and porcine pancreatic elastase (Figure 5, Table 2). Compound **1** exhibited 11-fold and 33-fold reduced activity against chymotrypsin and elastase, respectively, indicating that the condensation of Ahp and Thr-1 considerably deactivated largamide D (**2**) and to different extents for both enzymes tested.

### 2.4. Conclusion

Marine cyanobacteria produce secondary metabolites that function as chemical defenses against consumers. Many of these compounds are serine protease inhibitors that could be responsible for the antifeedant activity observed for *L.* cf. *confervoides* from Fort Lauderdale reefs. Future studies should show the contribution of each serine protease inhibitor or other secondary metabolite produced by this particular collection of *L.* cf. *confervoides* to the observed antifeedant activity of the extracts. Furthermore, the isolation of compound **1** suggests that intramolecular condensation can modulate the inhibitory activity. The Ahp moiety is oftentimes critical for protease inhibition, and structural and conformational changes involving this unit are expected to affect activity. If indeed biosynthetically driven, this represents an endogenous pathway to modulate enzymatic activities. In turn, if this process is reversible it could unlock a more potent inhibitor.

## 3. Experimental Section

### 3.1. General Experimental Procedures

^1^H and 2D NMR spectra for largamide D oxazolidine were acquired in DMF-*d*_7_ or MeOH-*d*_4_ on a Bruker 600 MHz spectrometer using residual solvent signals as the internal standard (*δ*_H_ 8.02, *δ*_C_ 162.3 for DMF-*d*_7_ and *δ*_H_ 3.30 *δ*_C_ 49.0 for MeOH-*d*_4_). HSQC experiments were optimized for ^1^*J*_CH_ = 145 Hz, and HMBC experiments were optimized for *^n^J*_C,H_ = 7 Hz. ^13^C NMR experiments were performed on a Bruker 500 MHz spectrometer (5 mm probe). HRMS data were obtained using an Agilent LC-TOF mass spectrometer equipped with an APCI/ESI multimode ion source detector, and low resolution mass spectra were obtained on a A3200 Q TRAP LC/MS/MS (hybrid triple quadrupole linear ion trap mass spectrometer, Applied Biosystems, USA) with an electrospray ionization (ESI) interface operated in positive mode. HPLC–based compound isolation was performed on a Shimadzu LC-20AT prominence LC system with peak detection by a Shimadzu SPD-M20A prominence diode array detector.

### 3.2. Marine Cyanobacterial Samples

Largamide D oxazolidine (**1**) was isolated from *L.* cf. *confervoides* samples collected at approximately 15 m depth from reefs near the Port Everglades Inlet, Fort Lauderdale, Florida, USA (26°05.9902′N, 80°05.0184′W) in August 2004 and May and August 2005. Largamide D (**2**) was isolated from *L.* cf. *confervoides* samples collected off the coast of Broward County (Fort Lauderdale and Pompano Beach, Florida, USA) (26°01.1414′N, 80°05.9973′W; 26°15.134′N, 80°03.908′W) at a depth of 7–15 m in July 2004 and August 2005. S. Golubic identified the cyanobacterium [20] and its 16S rDNA gene sequence has been reported [10,20].

### 3.3. Feeding Experiments

The fresh *L.* cf. *confervoides* collected from Broward County was returned to the laboratory and frozen immediately and then freeze-dried. The freeze-dried material was weighed and extracted three times in 1:1 ethyl acetate–methanol (non-polar) and then three times in 1:1 ethanol–water (polar). Each solvent mixture was left on the cyanobacterium for 24 hours and then exchanged for fresh solvent. Each of the three extracts of the same solvents were pooled and dried down under vacuum.

Fish feeding experiments were conducted with a natural assemblage of reef fish in the field at Golden Reef (7 m depth) in Belize (GPS: N 16° 48.575, W 088° 05.138) as previously described [25]. This site was selected because the reef fish assemblages were representative of Florida and Caribbean coral reefs, our methods were well worked out at this site and large numbers of tropical reef fishes would feed during the assay. Feeding experiments were conducted with agar-based food, composed of 5 g of fish food (Kent Platinum Reef Herbivore), 1.25 g of agar, 1.25 g of carrageenan, and mixed with 100 mL of boiling water. This mixture was poured warm into 1 cm^3^ molds and allowed to cool. The two treatment foods were made by incorporating a natural concentration of the extract (polar or non-polar) dissolved in 5 mL of ethanol into the agar mixture just before it was poured. Solvent only (5 mL of ethanol) was added to the control food. Most of the ethanol evaporated as the agar cooled and gelled. Four agar food cubes were attached with safety pins to each polypropylene rope and offered to fish in groups of three ropes (two treatments (one with nonpolar extract and one with polar extract incorporated at natural dry mass concentrations) and one control). Nineteen (19) replicate groups of three ropes were distributed around the reef. Fish including *Thalassoma bifasciatum*, *Scarus iserti*, and *Acanthurus chirurgus* were observed feeding on the food cubes. In the field the number of agar cubes eaten was recorded. Results were analyzed with Friedman’s random block test followed by a post-hoc multiple comparisons using the Student Newman Keuls method.

Feeding experiments with the sea urchin *Diadema antillarum* were conducted in the laboratory at Carrie Bow Cay in Belize. Individuals of *D. antillarum* were collected from the reef flat adjacent to the island and maintained individually in 5 gallon buckets with flow through seawater delivered to each bucket. The urchins were fed a small amount of *Padina* sp. before the experiment to ensure that they were not starved. The urchins were offered *L.* cf. *confervoides* extracts incorporated into artificial food that consisted of 2 g of dried and powdered *Gracilaria* and 1 g of agar. The dried ingredients were mixed with 40 mL of boiling water and either the polar or non-polar extract dissolved in 5 mL of ethanol or the ethanol alone was added to the agar mixture while it was cooling. The artificial food was poured as 2 × 35 cm strips on a sheet of window screen. The window screen was cut so that each urchin was offered a 2 × 3 cm strip of each food type, each of which covered 120 squares of the screen. The food was left with each urchin for approximately 24 hours. At the end of the experiment the number of window screen squares that were no longer covered by the agar food was counted. If the food was completely consumed or not consumed at all, that replicate urchin was not included in the statistical analysis. The number of squares eaten was recorded for each food type and was statistically compared using a Friedman’s random block test, followed by a post-hoc multiple comparisons using the Student Newman Keuls method.

### 3.4. Extraction and Isolation

The extraction and initial fractionation procedures of *L.* cf. *confervoides* samples collected from Port Everglades Inlet, Fort Lauderdale, Florida, are described in the isolation of tiglicamides [33]. The tiglicamide-rich fractions [33] were collected and subjected to repeated semi-preparative reversed-phase HPLC (Phenomenex Synergi 4 μm Hydro-RP, 250 × 10 mm, 2.0 mL/min; PDA detection at 200–400 nm) using two sequential linear gradients of MeOH in 0.05% aqueous TFA (60–90% over 25 min, 90–100% over 10 min) to furnish mixtures containing **1** and tiglicamides A–C. The final purification of **1** was achieved by using a Phenomenex Luna Phenyl-hexyl column 250 × 10 mm while maintaining the same HPLC conditions described above (**1**, *t*_R_ 25.5 min; 6.7 mg). The extraction and isolation of *L.* cf. *confervoides* samples collected from Broward County and Pompano Beach, Florida, are described in the isolation of lynbyastatin 4 [21]. The fraction eluting with 25% aqueous MeOH off a C_18_ Alltech SPE cartridge [21] was then purified by semi-preparative reversed-phase HPLC (YMC-Pack ODS-AQ, 250 × 10 mm, 2.0 mL/min; UV detection at 220 and 240 nm) using a MeOH–H_2_O linear gradient (20–100% over 70 min and then 100% MeOH for 10 min), to furnish largamide D (**2**, *t*_R_ 53.0 min; 2 mg).

### 3.5. Largamide D oxazolidine (**1**)

Colorless amorphous solid; [α]^20^_D_ –35 (*c* 0.1, MeOH), UV (MeOH) *λ*_max_ (log ɛ) 204 (4.23), 214 (sh) (4.02), 230 (sh) (3.71), 280 (3.22) nm; NMR data, see Table 1; HRESI/APCIMS *m/z* [M + Na]^+^ 1236.4745, 1238.4742 (ratio 1:1.2, calcd for C_56_H_80_^79^BrN_9_NaO_16_, 1236.4804; C_56_H_80_^81^BrN_9_NaO_16_, 1238.4784).

### 3.6. Protease Inhibition Assays

Elastase assay was assessed using high-purity porcine pancreatic elastase (Elastin Products Company, EC134, 135 units/mg). The assay buffer used was 1M Tris-HCl (pH 8.0). Assay buffer (79 μL), elastase solution (75 μg/mL in assay buffer, 5 μL) and various concentrations of **1** and **2** (1 μL, dissolved in DMSO) were pre-incubated for 15 min at room temperature in a microtiter plate. After this time, 15 μL of substrate solution (2 mM *N*-succinyl-Ala-Ala-Ala-*p*-nitroanilide in assay buffer) was added to each well and the reaction was followed by measuring the absorbance at 405 nm every 30 s. Inhibition of chymotrypsin was determined using the α-chymotrypsin from bovine pancreas (Sigma, C4129, 55 units/mg). The assay buffer was 50 mM Tris-HCl, 100 mM NaCl and 1 mM CaCl_2_ (pH 7.8). Assay buffer (39 μL), enzyme solution (100 μg/mL in assay buffer, 10 μL), and various concentrations of **1** and **2** (1 μL, dissolved in DMSO) were pre incubated for 30 min at room temperature before substrate solution (1.5 mM *N*-succinyl-Gly-Gly-Phe-*p*-nitroanilide in assay buffer) was added. The reaction was followed by measuring the absorbance at 405 nm every 30 s for 30 min. For each assay, enzyme activity of each well was calculated using the initial slope of each progress curve, expressed as a percentage of the slope of uninhibited reaction. All assays were carried out in triplicate at ambient temperature (*T* = 29 ºC). Molassamide, which also causes complete enzyme inhibition, was used as a positive control for inhibition of elastase (IC_50_ 32 nM) and chymotrypsin (IC_50_ 234 nM) [12]. Dose–response curve fitting was done using Xlfit Excel, MathlQ Version 2.2.2 (IDBS Ltd.).

## Figures and Tables

**Table 1:** NMR spectral data for the major conformer of largamide D oxazolidine (**1**) and largamide D (**2**) [24].

Unit^a^		1 (DMF-*d*_7_) *δ*_H_ (*J* in Hz)	*δ*_C_	COSY	HMBC	1 (methanol-*d*_4_) *δ*_H_ (*J* in Hz)	*δ*_C_	2 (methanol-*d*_4_) *δ*_H_ (*J* in Hz)	*δ*_C_
Val-1	1		171.4				173.6		174.8
	2	4.53, m	56.8	H-3, NH	1, 3, 4, 5, 1 (*N-*Me-Br-Tyr)	4.72, m	58.1	4.49, d (7.4)	58.9
	3	2.32, m	31.1	H-2, H_3_-4, H_3_-5	2, 4, 5	2.38, m	32.7	2.15, m	31.8
	4	0.92, d (7.0)	19.0	H-3	2, 3, 5	0.92, d (7.2)	18.8	0.93, d (6.9)	18.9
	5	0.86, d (7.0)	17.5	H-3	2, 3, 4	0.89, d (7.2)	18.0	0.99, d (6.9)	19.8
	NH	6.76, d (8.3)		H-2					
*N-*Me- Br-Tyr	1		169.2				170.9		171.4
	2	5.71, dd (11.5, 2.5)	62.5	H-3a/3b	1	5.75, dd (11.5, 2.4)	63.9	5.06, dd (11.5, 3.1)	62.6
	3a	3.34, dd (−14.3, 11.5)	32.4	H-2, H-3b H-2, H-3^a^	2, 4	3.28, dd (−14.4, 2.4)	33.5	3.40, dd (−14.6, 3.1)	33.8
	3b	2.89, dd (−14.3, 2.5)				2.84, dd (−14.4, 11.5)		2.80, m	
	4		130.5				131.4		130.4
	5	7.44, d (1.9)	133.8		4, 6, 7, 9	7.32, d (1.2)	134.8	7.34, d (2.0)	134.8
	6		109.3				111.2		110.8
	7		153.6				154.7		154.4
	8	7.03, d (8.2)	116.7	H-9	4, 6, 7, 9	6.83, d (8.3)	117.7	6.87, d (8.2)	117.6
	9	7.13, dd (8.2, 1.9)	129.9	H-8	5, 8	7.02, dd (8.3, 1.2)	130.8	7.17, dd (8.2, 2.0)	130.7
	*N*-Me	2.92, s	30.8		1, 2, 1 (Thr-1)	2.90, s	31.8	2.87, s	31.2
Thr-1^a^	1		169.7				172.0		173.0
	2	4.04, d (3.4)	59.5	H-3	1, 3, 2 (Ahp), H-6 (Ahp)	3.96, d (2.3)	61.0	4.57, d (6.8)	55.9
	3	4.36, qd (6.4, 3.4)	76.4	H_3_-4	1, 2, 4	4.38, dq (6.2, 2.3)	77.4	3.74, m	66.6
	4	0.71, d (6.4)	19.4	H-3	2, 3	0.70, d (6.2)	20.5	0.57, d (6.2)	19.6
Ahp^a^	2		168.8				170.5		171.3
	3	4.63, ddd (12.4, 8.7, 4.9)	50.3	H-4a/4b, NH	2, 4, 5, 6	4.63, dd (9.9, 5.3)	51.7	4.64, dd (12.4, 6.4)	50.7
	4a	1.95, m	24.6	H-3, H-4b, H-5a/5b	2, 3, 5, 6	1.97, m	25.3	2.82, m	22.0
	4b	1.88, m		H-3, H-4a, H-5a/5b		1.93, m		1.88, m	
	5a	2.38, m	27.9	H-4a/4b, H-5b, H-6	3, 4, 6	2.40, m	29.2	2.04, m	30.6
	5b	1.68, m		H-4a/4b, H-5a, H-6		1.66, m		1.87, m	
	6	5.01, dd (8.5, 6.2)	87.4	H-5a/5b	2, 4, 5	5.03, dd (8.5, 6.5)	88.6	5.33, brs	76.9
	NH	7.22, d (8.7)		H-3	3, 1 (Leu)				
Leu	1		171.7				174.6		174.4
	2	4.49, m	51.4	H-3a/3b	1, 3	4.58, dd (11.0, 3.0)	52.6	4.56, dd (8.5, 3.3)	52.9
	3a	1.84, m	40.6	H-2, H-3b	1, 2, 4	1.88, m	41.3	1.98, ddd (12.6, 12.6, 3.3)	40.2
	3b	1.47, m		H-2, H-3a		1.47, m		1.55, m	
	4	1.61, m	24.5	H-3a/3b, H_3_-5, H_3_-6	2, 3, 5, 6	1.56, m	25.8	1.65, m	25.8
	5	0.87, d (6.6)	22.9	H-4	4	0.91, d (6.0)	23.8	0.87, d (6.8)	20.0
	6	0.82, d (6.6)	20.8	H-4	4	0.83, d (6.0)	21.5	0.97, d (6.7)	23.7
	NH	8.50, d (8.5)		H-2	2, 1 (Thr-2)				
Thr-2	1		169.7				171.1		170.9
	2	4.92, dd (9.0, 1.7)	55.4	NH	1, 3, 1 (Val-2)	4.72, m	56.7	4.78, d (1.2)	56.3
	3	5.78, qd (6.0, 1.7)	72.1	H_3_-4	1, 2, 4, 1 (Val-1)	5.64, qd (6.5, 1.4)	73.2	5.59, qd (6.5, 1.2)	73.4
	4	1.33, d (6.0)	16.6	H-3	2, 3	1.30, d (6.5)	17.7	1.36, d (6.5)	18.1
	NH	8.25, d (9.0)		H-2	2, 1 (Val-2)				
Val-2	1		171.7				173.8		173.9
	2	4.53, dd (8.6, 5.5)	58.0	H-3, NH	1, 3, 4, 5	4.30, d (7.8)	60.2	4.39, d (7.4)	59.7
	3	2.17, m	31.2	H-2, H_3_-4, H_3_-5	2, 4, 5	2.09, dd (7.8, 6.8)	32.0	2.17, m	31.9
	4	0.90, d (7.0)	18.5	H-3	2, 3, 5	0.94, d (6.8)	18.8	0.98, d (6.9)	18.5
	5	0.87, d (7.0)	17.5	H-3	2, 3, 4	0.90, d (6.8)	19.4	0.98, d (6.9)	19.8
	NH	7.76, d (8.6)		H-2	2, 1 (Ala)				
Ala	1		172.0^b^				174.8		174.4
	2	4.49, dq (9.0, 7.2)	48.9	H-3, NH	1, 3	4.38, q (7.0)	49.0	4.45, m	49.9
	3	1.30, d (7.2)	17.0	H-2	1, 2	1.33, d (7.0)	17.8	1.38, d (7.2)	17.6
	NH	8.26, d (9.0)		H-2	2, 1 (Ahppa)				
Ahppa	1		171.9^b^				174.1		173.5
	2	4.52, m	52.6	H-3a/3b, NH	1, 3, 4, NH	4.41, m	54.4	4.46, m	53.9
	3a	1.89, m	32.4	H-2, H-3b, H-4a/4b	2, 4, 5	1.87, m	32.7	1.91, m	32.6
	3b	1.73, m		H-2, H-3a, H-4a/4b		1.69, m		1.73, m	
	4a	1.68, m	28.2	H-3a/3b, H-4b	2, 3, 5, 6	1.68, m	29.0	1.70, m	28.8
	4b	1.62, m		H-3a/3b, H-4a		1.63, m		1.66, m	
	5	2.52, m	34.2	H-4a/4b	3, 4, 6	2.54, m	35.6	2.57, t (7.3)	35.2
	6		132.6				134.0		133.6
	7/11	7.03, d (8.3)	129.8	H-8/10	6, 8/10, 9	7.00, d (8.4)	130.4	7.02, dd (8.2, 1.8)	130.2
	8/10	6.76, d (8.3)	115.2	H-7/11	7/11, 9	6.67, d (8.4, 2.4)	116.1	6.70, dd (8.2, 1.8)	115.8
	9		156.0		7/11, 8/10		156.5		156.2
	9-OH	9.36, s			8/10, 9				
	NH	7.87, d (7.6)		H-2	1, 2, 1 (Ga)				
Ga	1		172.4				175.4		174.9
	2	4.11, m	73.5	H-3a/3b	1, 3	4.11, d (3.5)	74.2	4.13, t (3.8)	73.9
	2-OH	c							
	3a	3.76, dd (−10.9, 3.5)	64.7	H-2, H-3b	1, 2	3.78, 2H, d (3.5)	65.6	3.79, 2H, d (3.8)	65.2
	3b	3.70, dd (−10.9, 5.6)		H-2, H-3a					

a The initial numbering from largamide D. (**2**) has been retained for comparison of data [24].

b Can be interchanged.

c Not observed.

**Table 2:** Serine protease inhibitory activities (IC_50_, μM) of compounds **1** and **2**.

	Chymotrypsin	Elastase
Largamide D (**2**)	0.083 ± 0.008	0.045 ± 0.003
Largamide D oxazolidine (**1**)	0.928 ± 0.093	1.52 ± 0.08
